# Case Report: A rare case of biopsy-proven lupus nephritis complicated by Graves’ disease in a 39-year-old man

**DOI:** 10.3389/fimmu.2026.1908289

**Published:** 2026-10-28

**Authors:** Xichang Wang, Xiaoguang Shi, Beiru Zhang, Mi Tian

**Affiliations:** 1 Department of Endocrinology, Shengjing Hospital of China Medical University, Shenyang, Liaoning, China; 2 Department of Nephrology, Shengjing Hospital of China Medical University, Shenyang, Liaoning, China

**Keywords:** autoimmune thyroid diseases, case reports, Graves’ disease, hyperthyroidism, lupus nephritis, systemic lupus erythematosus

## Abstract

Hyperthyroidism and systemic lupus erythematosus (SLE) are both common diseases in internal medicine. Among them, Graves’ disease (GD) is the most frequent cause of hyperthyroidism, while lupus nephritis (LN) represents one of the most severe organ manifestations of SLE. Basic research suggests that GD and SLE may share overlapping genetic and immunological pathways; however, whether these shared mechanisms contribute to their clinical coexistence remains uncertain. Clinical studies or case reports on their coexistence are currently limited, and we cannot effectively manage cases of co-occurrence based on authoritative guidelines or expert consensus. In this study, we report a 39-year-old male patient with biopsy-proven ISN/RPS Class IV + V LN concomitant with GD. The patient was treated with a combination of glucocorticoids, mycophenolate mofetil, and hydroxychloroquine for LN, along with methimazole for GD, supplemented by symptomatic therapy including antihypertensive and heart rate controlling agents, etc. The patient demonstrated early clinical and laboratory improvement during follow-up, although the limited follow-up precluded assessment of an established renal response.

## Introduction

Graves’ disease (GD) is the most common cause of hyperthyroidism and is characterized by thyroid-stimulating hormone receptor autoantibody (TRAb) - mediated thyroid dysfunction. Systemic lupus erythematosus (SLE) is a chronic multisystem autoimmune disease involving dysregulation of both humoral and cellular immunity. Increasing evidence suggests that autoimmune thyroid diseases (AITD) and SLE share common genetic susceptibility loci and immunological pathways, including aberrant B-cell activation, autoantibody production, and cytokine-mediated immune responses (1). A meta-analysis published by our research group indicated that the titer of thyroid autoantibodies in SLE patients was significantly higher than that in controls (2). Consequently, several retrospective cohort studies in recent years have suggested that GD is an independent risk factor for SLE (3–5), and vice versa (6). A Mendelian randomization analysis implied a potential causal association between the two diseases (7). However, previous research findings regarding the association between GD and SLE are not entirely consistent. The positive association between the two has also been denied in several studies (8–10). Therefore, more large-sample cohort studies are required to verify the potential association between the two.

The concurrence of GD and lupus nephritis (LN), one of the most severe organ manifestations of SLE, remains uncommon in clinical practice. The coexistence of these conditions may complicate diagnostic evaluation and therapeutic decision-making because hyperthyroidism can influence renal hemodynamics and immune activity, while the treatment for LN may affect the diagnosis and management of thyroid dysfunction (11–14). Currently, there are no authoritative guidelines or expert consensus to guide clinical decision-making due to the rarity of this comorbidity. Herein, we report a 39-year-old male patient with GD complicated by LN and review the relevant evidence to improve clinicians’ understanding of this rare clinical comorbidity.

## Case presentation

A 39-year-old man was admitted to our hospital with fatigue and bilateral eyelid edema for two weeks, and newly detected proteinuria for five days. The patient initially developed upper respiratory symptoms, including headache and nasal congestion, without fever or cough. He self-medicated with compound paracetamol and cefalexin without improvement. He subsequently experienced left abdominal bloating, nausea, and occasional postprandial vomiting. Laboratory evaluation at a local hospital revealed hypoalbuminemia (albumin 21 g/L), anemia (hemoglobin 106 g/L), thrombocytopenia (platelet 87×10^9^/L) and proteinuria before admission. Abdominal CT scan was unremarkable. The patient was referred to Shengjing Hospital of China Medical University for further evaluation.

**Table 1** shows several key time points in the patient’s medical process. At our outpatient clinic before admission, investigations showed significant proteinuria (4+), microscopic hematuria (occult blood 3+, RBC 32.49/HP), positive antinuclear antibody (ANA IgG), and positive anti-double-stranded DNA antibody (dsDNA IgG). Serum albumin was 20.2 g/L. The patient was admitted to the internal medicine ward of our hospital with a primary diagnosis of LN (highly likely).

**Table 1:** Timeline of the clinical course.

Relative time	Clinical events
Two weeks before admission	The patient developed symptoms of thyrotoxicosis and renal involvement. He self-medicated with compound paracetamol and cefalexin without improvement.
One week before admission	Laboratory evaluation at a local hospital revealed hypoalbuminemia, anemia, thrombocytopenia and proteinuria.
Two days before admission	The patient came to our internal medicine outpatient clinic for treatment. Investigations showed significant proteinuria, microscopic hematuria, positive ANA IgG and positive dsDNA IgG.
Day 0 (admission)	The patient came to our internal medicine ward to be admitted. Laboratory and imaging evaluations were conducted. Antithyroid drugs, antihypertensive drugs, heart rate lowering drugs and stomach-protecting drugs were administrated.
Day 3	Given that laboratory test alone is insufficient to definitively diagnose LN, the patient was scheduled for kidney biopsy.
Day 4	Biopsy was reported and ISN/RPS Class IV + V LN was confirmed. Glucocorticoids, mycophenolate mofetil, hydroxychloroquine and other supportive treatments were initiated.
Day 5	The treatment plan was determined, and the patient was scheduled for discharge.
Three weeks after discharge	The patient returned to the outpatient clinic for a follow-up examination. Laboratory test results, such as blood routine, liver function, kidney function and urinary protein, were trending towards improvement.
One month after discharge	The patient participated in a clinical trial on tacrolimus for the treatment of LN.

LN, lupus nephritis; ISN, International Society of Nephrology; RPS, Renal Pathology Society; ANA, anti-nuclear antibody.

On review of systems, he reported persistent left abdominal bloating, mild right knee pain, hair loss, and increased foamy urine. He denied fever, cough, dyspnea, chest pain, gross hematuria, melena, rash, oral ulcers, sicca symptoms or photosensitivity. The patient reports recent increased appetite, poor sleep quality, and a weight loss of approximately 2.5 kg over the past month.

After admission, further questioning was conducted regarding past medical history. The patient reported a history of chronic gastritis of several years, characterized by occasional postprandial epigastric fullness without reflux or heartburn, and no regular medication. He denied hypertension, diabetes, coronary artery disease, hepatitis, tuberculosis, or other chronic or infectious diseases. Six months prior, he sustained a right upper limb fracture treated with external fixation at a local hospital, without surgical reduction. No other surgeries, trauma, or blood transfusions were reported. He also reported intermittent headaches over the past 3 years, occasionally treated with analgesics.

On admission, the patient was alert and oriented. Vital signs were as follows, temperature 36.5 °C, heart rate 108 beats/min, respiratory rate 18 breaths/min, and blood pressure (BP) 148/96 mmHg. He appeared pale, with bilateral eyelid and lower limb edema. Thyroid examination revealed a grade II goiter with audible vascular bruit. Fine tremor was noted in both hands. No other abnormal findings were observed on physical examination.

As shown in **Tables 2**, **3**, the patient underwent routine laboratory and imaging evaluations. Thyroid function tests revealed a suppressed thyroid stimulating hormone (TSH) below the detectable limit, elevated free triiodothyronine (FT3) and free thyroxine (FT4), and positive TRAb. Thyroid ultrasonography demonstrated diffuse thyroid enlargement with heterogeneous echotexture and markedly increased vascularity, consistent with a diagnosis of GD. Electrocardiography showed sinus tachycardia.

**Table 2:** Results of main laboratory tests of this patient at outpatient and after admission.

Indicator	Unit	Reference value	Result
Thyroid stimulating hormone (TSH)	mIU/L	0.35-4.75	<0.0051
Free triiodothyronine (FT3)	pmol/L	3.50-7.00	12.53
Free thyroxine (FT4)	pmol/L	10.00-22.00	37.69
Thyroid peroxidase antibody (TPOAb)	IU/mL	0-30	340.11
Thyroglobulin antibody (TgAb)	IU/mL	0-75	105.76
TSH receptor antibody (TRAb)	IU/L	0-1.75	8.31
Anti-nuclear antibody (ANA) IgG	–	Negative	Positive
Anti-nucleosome IgG antibody	–	Negative	Positive
Anti–double-stranded DNA (dsDNA) IgG antibody	–	Negative	Positive
Albumin (ALB)	g/L	40-55	20.2
Alanine aminotransferase (ALT)	U/L	9-50	18
Aspartate aminotransferase (AST)	U/L	15-40	24
Direct bilirubin	μmol/L	0-6.8	4.0
Unconjugated bilirubin	μmol/L	0-19.2	6.5
Creatinine	μmol/L	57-97	82
Uric acid (UA)	μmol/L	208-428	406
Glucose (Glu)	mmol/L	3.89-6.11	5.87
Triglyceride (TG)	mmol/L	0-1.7	0.91
Total cholesterol (TC)	mmol/L	0-5.2	4.31
High density lipoprotein (HDL)	mmol/L	≥1.0	1.16
Low density lipoprotein (LDL)	mmol/L	<3.4	2.73
Brain natriuretic peptide (BNP)	ng/L	<100	102.23
Complement C3	g/L	0.80-1.60	0.61
Complement C4	g/L	0.20-0.40	0.06
C-reactive protein (CRP)	mg/L	0-6	27.56
Erythrocyte sedimentation rate (ESR)	mm/h	0-15	53
Urine α1-microglobulin (A1M)	mg/dL	0-1.20	8.69
Urine IgG	mg/dL	0-0.96	46.4
Urine N-acetyl-β-D-glycosaminidase	U/L	0-11.5	32.80
Urine Transferrin	mg/dL	0-0.20	22.42
Urine microalbumin	mg/L	≤30	3768.5
Red blood cell (RBC)	10^12^/L	4.3-5.8	3.74
Hemoglobin (Hb)	g/L	130-175	105
White blood cell (WBC)	10^9^/L	3.5-9.5	4.6
Platelet (PLT)	10^9^/L	125-350	103
Erythropoietin (EPO)	mIU/mL	2.59-18.50	26.22
Ferritin	ng/mL	23.9-336.2	672.09
Urine occult blood	–	Negative	3+
Urine protein	–	Negative	4+
Urine glucose	–	Negative	Negative
Urine RBC	/HP	0-3.03	149.58
Urine WBC	/HP	0-2.14	14.61
Urine hyaline cast	/μL	0-0.34	5.52
Urine pathologic cast	/μL	0-0.34	11.04
24-hour urine protein	g/d	0-0.15	11.44

**Table 3:** Results of imaging examinations of this patient after admission.

Physical Examination Items	Examination Results
Electrocardiogram	Sinus tachycardia
Echocardiography	Degenerative changes of the aortic valve with mild regurgitation, pericardial effusion (localized small amount, approximately 4 mm anterior to the right ventricle), and left ventricular systolic function at the lower limit of normal at rest (LVEF 53%).
Non-contrast chest CT	Scattered solid nodules in both lungs (largest diameter 6 mm), scattered enlarged lymph nodes in the mediastinum and bilateral axillae (largest short-axis diameter 10 mm), and a small amount of pericardial effusion.
Thyroid ultrasonography	Diffuse thyroid enlargement with increased vascularity, and bilateral cervical lymphadenopathy (largest node measuring 3.1×1.8×0.9 cm).
Urinary system ultrasonography	Enlarged prostate with calcifications (prostate measuring approximately 3.1×4.9×3.3 cm; the largest calcification measuring about 0.5 cm in diameter).

Autoimmune serology confirmed positive antinuclear antibody (ANA), anti- nucleosome antibody, and anti-double-stranded DNA (dsDNA) antibody, supporting a diagnosis of systemic lupus erythematosus (SLE), while other components of the ANA panel were negative. Serum complement levels were also reduced, with C3 and C4 levels of 0.61 g/L and 0.06 g/L, respectively. Serum albumin was notably decreased, whereas liver function tests, including transaminases and bilirubin, were within reference intervals. Renal function, uric acid, blood glucose, and lipid profile were unremarkable.

Cardiac assessment showed mildly elevated B-type natriuretic peptide (BNP), and echocardiography demonstrated preserved left ventricular systolic function at the lower end of normal (LVEF 53%) with a small pericardial effusion. Inflammatory markers, including C-reactive protein and erythrocyte sedimentation rate, were elevated.

Hematologic evaluation revealed mild anemia (hemoglobin 105 g/L) and mild thrombocytopenia (platelet 103×10^9^/L), with normal leukocyte counts. Urinalysis showed significant hematuria and proteinuria. Urinary sediment analysis revealed increased numbers of hyaline and pathological casts, suggesting notable kidney involvement. Several markers reflecting renal tubular function were also significantly increased, as shown in **Table 2**.

Based on the patient’s medical history, physical examination, and auxiliary laboratory and imaging findings, the preliminary diagnoses were GD, SLE, LN (highly likely), cardiac insufficiency, hypertension, sinus tachycardia, mild anemia, mild thrombocytopenia, and hypoalbuminemia. Based on the preliminary diagnosis above, the patient was given methimazole 10 mg orally three times daily to treat GD. The patient had hypertension with borderline ventricular systolic function and mild pericardial effusion, so sacubitril/valsartan 50 mg orally twice daily was given for BP control. Additionally, propranolol 10 mg orally three times daily was given to control the heart rate. The above medications were supplemented with omeprazole 40 mg orally once daily to protect the gastric mucosa. Given that laboratory test alone is insufficient to definitively diagnose LN, the patient was immediately scheduled for an ultrasound-guided kidney biopsy on Day 3 after admission.

Light microscopic examination of four renal cortical biopsy cores (0.4–0.6 cm in length) identified 35 glomeruli, of which at least 20 (≥57.1%) were affected by proliferative lesions, supporting the diagnosis of diffuse LN (ISN/RPS Class IV). None of the glomeruli exhibited global or segmental sclerosis. The glomeruli demonstrated mild-to-moderate mesangial hypercellularity, segmental endocapillary proliferation with leukocyte infiltration and karyorrhexis, diffuse thickening of the glomerular basement membrane with spike formation, focal double-contour formation, two cellular crescents, and one fibrocellular crescent. Tubular epithelial cells showed vacuolar and granular degeneration, accompanied by occasional proteinaceous casts. Mild focal interstitial inflammatory cell infiltration was present without significant interstitial fibrosis, while the arterial structures appeared unremarkable. Immunofluorescence microscopy revealed granular immune-complex deposition along the glomerular capillary walls and within the mesangium, with positive staining for IgG (+++), IgG1 (+++), IgG3 (+++), IgA (++), C3 (++), C1q (+++), IgM (+), Kappa (+++), and Lambda (+++). Based on these findings, the pathological diagnosis was diffuse proliferative and membranous LN (ISN/RPS, Class IV + V), with an NIH Activity Index (AI) of 10/24 and a Chronicity Index (CI) of 2/12. Representative immunofluorescence findings are shown in **Figure 1A**, and light microscopic images obtained using hematoxylin–eosin (HE), periodic acid–Schiff (PAS), periodic acid–silver methenamine (PASM), and Masson’s trichrome staining are presented in **Figure 1B**. Electron microscopic examination was performed on two glutaraldehyde-fixed renal cortical tissue fragments (**Figure 2**), in which two glomeruli were evaluated. Segmental endocapillary hypercellularity with endothelial cell vacuolar degeneration was observed. Aggregated erythrocytes were present within some capillary lumina, resulting in luminal narrowing and compression. The glomerular basement membranes exhibited irregular thickening, measuring up to 800 nm, with focal wrinkling and mild subendothelial widening. Podocytes demonstrated swelling, vacuolar degeneration, and extensive foot process effacement. Mild-to-moderate mesangial hypercellularity and matrix expansion were also noted. Electron-dense deposits were identified in the subepithelial, intramembranous, mesangial, and focal subendothelial regions. In addition, tubular epithelial cells exhibited vacuolar degeneration with focal detachment, accompanied by mild interstitial inflammatory cell infiltration. Collectively, the ultrastructural findings, together with the light microscopic and immunofluorescence features, confirmed the diagnosis of proliferative and membranous LN (ISN/RPS Class IV + V).

**Figure 1:**
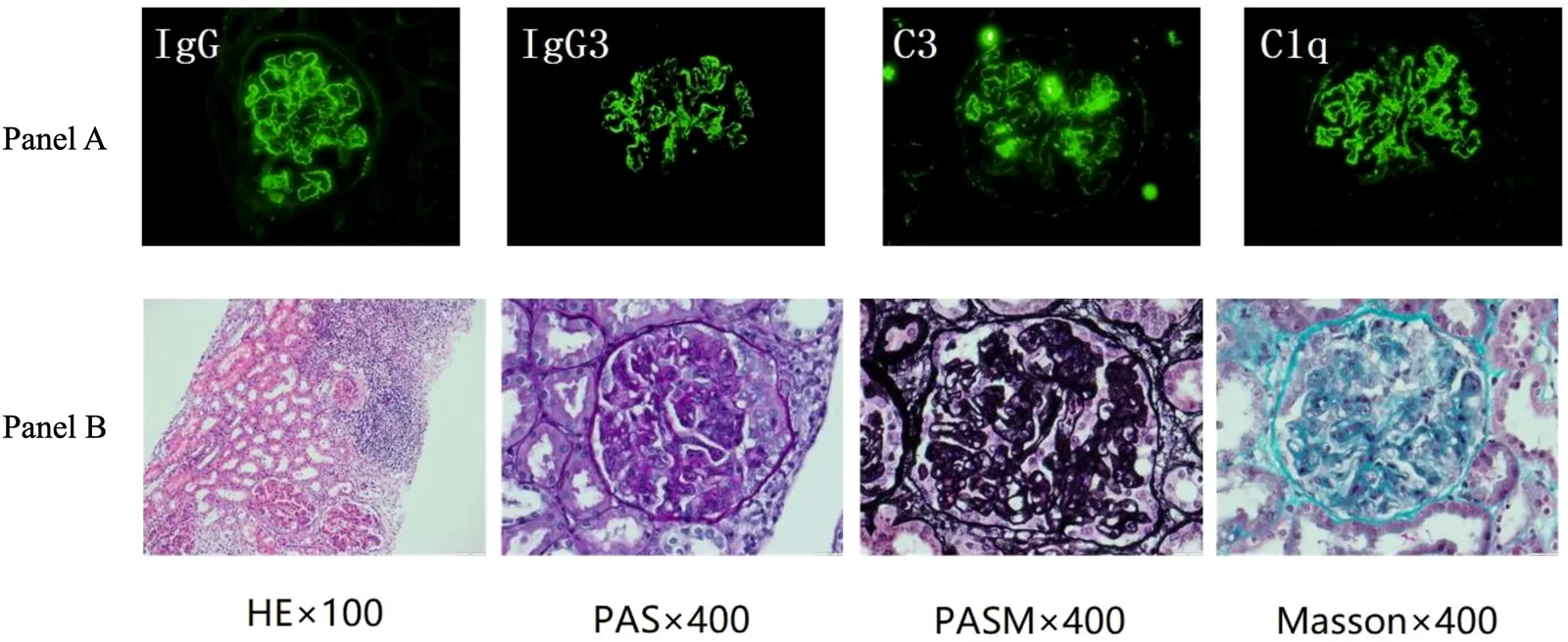
Renal biopsy findings on light microscopy and immunofluorescence. **(A)** Immunofluorescence microscopy shows granular deposition of IgG, IgG3, C3, and C1q in glomeruli, suggestive of ISN/RPS Class IV+V LN. **(B)** Light microscopy (HE, PAS, PASM and Masson) shows diffuse proliferative changes with mesangial and endocapillary hypercellularity and thickening of glomerular capillary walls, suggestive of ISN/RPS Class IV+V LN.

**Figure 2:**
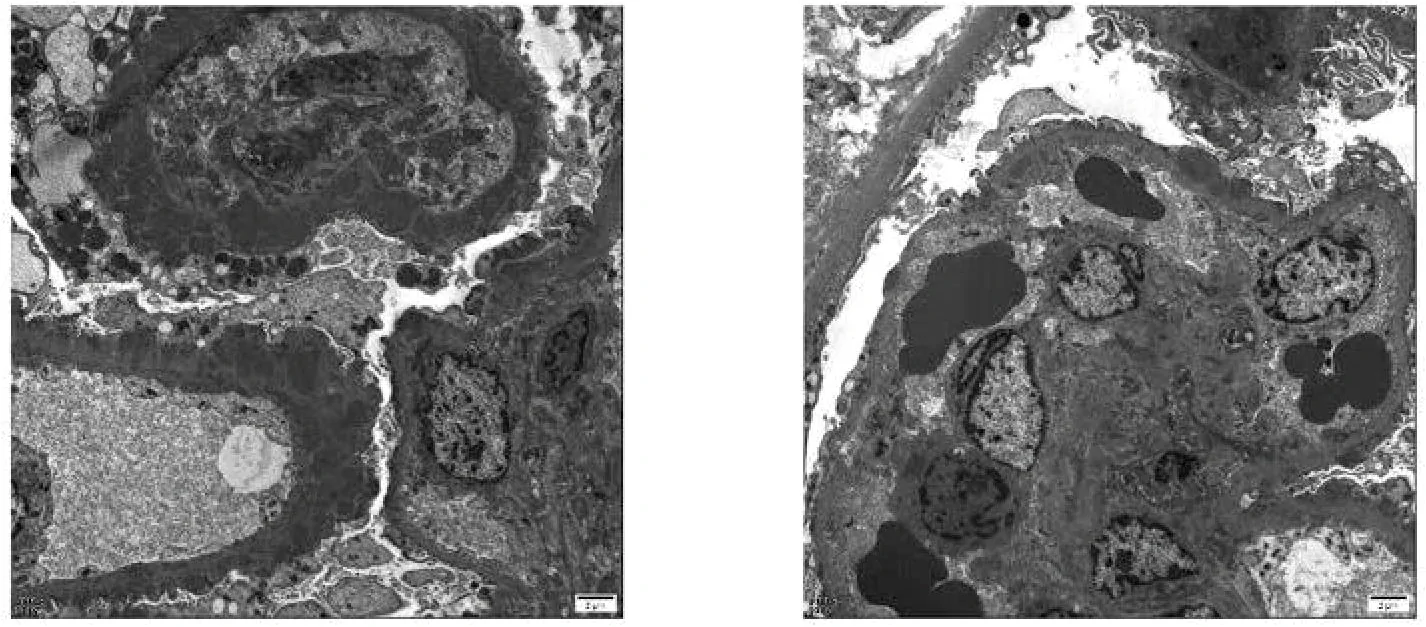
Electron microscopy of renal biopsy specimen. Electron microscopy shows electron-dense deposits in mesangial, subendothelial, and subepithelial areas of the glomerular basement membrane, with podocyte foot process effacement, supporting immune complex–mediated glomerular injury. The scale bars represent 2 μm.

Furthermore, antiphospholipid antibody testing demonstrated a positive lupus anticoagulant, with an elevated dilute Russell’s viper venom time (dRVVT) screening ratio of 1.47 (reference <1.20) and a normalized dRVVT ratio of 1.34 (reference <1.20). Anticardiolipin antibodies (IgG, IgM, and IgA) were negative. Anti-β2 glycoprotein I (anti-β2GPI) IgM and IgA were also negative, whereas anti-β2GPI IgG was markedly elevated at 99.8 CU (reference < 20 CU). Given that antiphospholipid antibodies are frequently detected in patients with SLE and may contribute to renal vascular injury and adverse renal outcomes, the coexistence of lupus anticoagulant and high-titer anti-β2GPI IgG was considered clinically relevant in the subsequent treatment. Further ultrasonography of the bilateral lower extremities ruled out the possibility of deep-vein thrombosis.

Given the possible initiation of intensive immunosuppressive therapy, infectious and malignant etiologies were considered. Screening tests for hepatitis, HIV and syphilis were negative, and the PPD skin test performed 72 hours after admission was also negative. Serum tumor markers (including CEA, CA125, CA199, AFP) were within the normal ranges. Respiratory medicine and thoracic surgery specialists were consulted, and no immediate invasive diagnostic intervention was recommended based on the overall clinical presentation and imaging findings. Close clinical and radiological follow-up was advised. Based on the renal biopsy pathology results, with the patient’s informed consent, we started administering methylprednisolone 250 mg once daily intravenously for three consecutive days. Subsequently, we started administering prednisolone acetate 60mg orally once daily and mycophenolate mofetil 0.75 g orally every 12 hours. After completing fundus photography and consultation with our hospital’s ophthalmology specialists, hydroxychloroquine 200 mg orally twice daily was added to the above medication regimen. In addition, the patient received oral calcium and vitamin D supplementation alongside glucocorticoid therapy. Given the presence of lupus anticoagulant and high-titer anti-β2GPI IgG, which constituted a high-risk antiphospholipid antibody profile, low-dose aspirin (100 mg once daily) was initiated as individualized primary thromboprophylaxis during the period of intensive immunosuppressive treatment. However, the patient had no history of thrombosis, and persistence of antiphospholipid antibodies at ≥12 weeks could not be established because of the limited follow-up. In addition, prophylaxis with trimethoprim–sulfamethoxazole (TMP 80 mg/SMX 400 mg orally) was initiated to reduce the risk of opportunistic infections during immunosuppressive therapy.

After determining the above treatment plan, the patient was scheduled for discharge and instructed to return to the outpatient clinic for a follow-up examination approximately three weeks after discharge. No significant adverse events were observed during the treatment. Repeat laboratory investigations at the three-week follow-up demonstrated an improvement in hematological parameters, with hemoglobin increasing to 125 g/L and platelet count recovering to the normal range (215×10^9^/L), while the leukocyte count remained within the reference interval. Serum albumin showed an increase to 25.0 g/L, whereas liver transaminases and bilirubin levels remained within the reference ranges. Renal parameters also improved following treatment. The serum creatinine level remained within the normal range. Notably, 24-hour urinary protein excretion decreased from 11.44 g/day at admission to 6.23 g/day, indicating an early reduction in proteinuria; however, the residual proteinuria remained clearly within the nephrotic range. The patient was advised to continue close outpatient follow-up with repeat laboratory assessments, including thyroid function, renal function and 24-hour urinary protein quantification to monitor therapeutic response and disease activity. Given that the existing financial resources were insufficient to support the patient’s subsequent follow-up examinations and treatment, the patient joined a clinical trial at our hospital on tacrolimus for the treatment of LN approximately one month after discharge. It is unknown whether the patient joined the treatment group or the placebo group.

## Discussion

The coexistence of GD and SLE is uncommon in clinical practice. Although both disorders are autoimmune diseases characterized by immune dysregulation and loss of self-tolerance, their simultaneous occurrence has been reported only sporadically. Previous studies have suggested that patients with AITD exhibit an increased risk of developing systemic autoimmune disorders, including SLE (15). To the best of our knowledge, biopsy-confirmed LN occurring concurrently with GD remains rarely reported. A previous report described Class IV LN associated with GD (16), indicating that the coexistence of these conditions is not unprecedented. Importantly, LN may also occur in the context of antithyroid drug-associated lupus or vasculitis, particularly after prolonged exposure to propylthiouracil (17–19). In contrast, our patient had no prior history of antithyroid drug exposure before presentation, and renal biopsy demonstrated ISN/RPS Class IV+V LN. Therefore, this case adds to the limited clinical literature describing the spontaneous coexistence of biopsy-proven LN and GD, particularly the Class IV+V pathological pattern. In patients presenting with GD accompanied by unexplained proteinuria, hematuria, edema, or renal dysfunction, clinicians should be aware of the possibility of concomitant autoimmune kidney disease rather than attributing all abnormalities solely to hyperthyroidism or antithyroid drug therapy.

Although individual cases cannot definitively establish the precise pathogenetic link between the two diseases, we must acknowledge that a substantial body of prior evidence suggests the existence of shared mechanisms. Current evidence suggests that shared genetic, immunologic, and environmental factors may contribute to the coexistence of these autoimmune conditions. Genetic susceptibility loci involved in immune regulation, such as HLA-DR3, CTLA-4, PTPN22, have been implicated in both conditions, predisposing individuals to loss of self-tolerance (20–25). Immunologically, breakdown of central and peripheral tolerance leads to persistent activation of autoreactive T cells, accompanied by impaired regulatory T-cell function and enhanced B-cell survival, partly mediated by increased BAFF signaling (26, 27). In SLE, a prominent type I interferon signature further amplifies antigen presentation and B-cell activation, which may facilitate the emergence of thyroid-directed autoimmunity (28). Conversely, thyroid autoantigens may serve as additional immunologic targets in a pro-inflammatory milieu, promoting epitope spreading. Imbalance between Th17 and Treg pathways, along with epigenetic modifications affecting immune-related gene expression, further contributes to chronic immune activation (29, 30). Environmental triggers such as infections, hormonal influences, and other external stressors may act as initiating or exacerbating factors (31, 32). Collectively, these observations provide a plausible biological framework for the coexistence of GD and SLE, but they should be interpreted as hypothetical mechanisms rather than evidence of a direct causal relationship between the two diseases. Whether shared genetic or immunological pathways contribute to the clinical coexistence of GD and LN requires further investigation in larger cohorts and mechanistic studies.

In the present case, the renal biopsy revealed Class IV+V LN according to the ISN/RPS classification. This combined histological pattern is characterized by diffuse proliferative lesions involving ≥50% of glomeruli together with membranous immune-complex deposition along the glomerular capillary walls. Class IV lesions are generally considered the most severe and aggressive form of LN, often associated with hematuria, proteinuria, renal dysfunction, and a higher risk of progression to chronic kidney disease if not treated promptly. The coexistence of Class V lesions may further contribute to nephrotic-range proteinuria and unfavorable renal outcomes. According to the updated KDIGO 2024 and EULAR 2025 recommendations, active proliferative LN, including mixed Class IV+V disease, requires prompt immunosuppressive therapy with glucocorticoids combined with steroid-sparing agents (33, 34). While mycophenolate mofetil and cyclophosphamide remain established induction therapies, recent guidelines have incorporated newer combination strategies involving biologic agents or calcineurin inhibitors. Hydroxychloroquine is recommended as background therapy for most patients with SLE due to its beneficial effects on disease control and relapse prevention. In our patient, renal biopsy confirmed Class IV+V LN with nephrotic-range proteinuria. Treatment with high-dose glucocorticoids, mycophenolate mofetil, and hydroxychloroquine was initiated, which was basically consistent with current guideline-based management of active LN. Following discharge, the patient was enrolled in a tacrolimus-related clinical trial approximately one month later; although the treatment allocation was unavailable, this reflected the evolving role of calcineurin inhibitors-based strategies in LN management. The patient showed early clinical and laboratory improvement, including recovery of cytopenias, an increase in serum albumin, and a reduction in proteinuria; however, these findings should be interpreted as early improvement rather than an established renal response because of the limited follow-up.

The presence of nephrotic-range proteinuria in patients with GD warrants careful evaluation, as several renal pathologies have been reported in association with GD, including membranous nephropathy and IgA nephropathy. Several representative case reports suggest that these associations may exist, but it has not been confirmed in large cohort studies considering that it is impractical to perform renal biopsy in large populations (35–38). In the present case, the coexistence of heavy proteinuria, hypocomplementemia, and positive anti-dsDNA antibodies strongly suggested LN, which was subsequently confirmed by renal biopsy. Histopathological examination demonstrated diffuse proliferative and membranous LN (Class IV+V), highlighting the indispensable role of renal biopsy in establishing an accurate diagnosis and guiding immunosuppressive therapy.

Another notable finding in this patient was the presence of lupus anticoagulant and high-titer anti-β2GPI IgG antibodies. Although no clinical thrombotic manifestations were observed, the presence of lupus anticoagulant and high-titer anti-β2GPI IgG indicates an increased thrombotic risk profile. Low-dose aspirin was therefore considered on an individualized basis in this patient, particularly in the setting of active SLE/LN and intensive immunosuppressive therapy. Nevertheless, a diagnosis of antiphospholipid syndrome could not be established because the patient had no documented clinical thrombotic event or persistence of antiphospholipid antibodies at ≥12 weeks. Longer-term follow-up with repeat antiphospholipid antibody testing would be required to further characterize his thrombotic risk.

Despite the insights provided by this case, several limitations should be acknowledged. First, the temporal relationship between LN and GD remains unclear, and current clinical evidence does not allow a causal link to be established. Second, as a single-case report, the findings may not be generalizable, given potential variability in disease presentation, progression, and treatment response among patients. Third, the use of sacubitril/valsartan was a non-standard aspect of the patient’s concomitant cardiovascular management and was not intended as LN-specific therapy; given the absence of an overt heart failure diagnosis, this treatment choice should be interpreted cautiously. Finally, it is regrettable that we were not able to follow up with this patient because the patient did not reside in Shenyang and joined a clinical trial one month after discharge. At the three-week outpatient follow-up, laboratory parameters showed early improvement, including recovery of cytopenias, an increase in serum albumin, and a reduction in proteinuria. However, these findings should be interpreted as early clinical and laboratory improvement rather than an established renal response because of the limited follow-up.

In conclusion, this case highlights the rare coexistence of biopsy-proven LN and GD. The case underscores the importance of maintaining a high index of suspicion for systemic autoimmune diseases in patients with AITD who develop unexplained proteinuria or multisystem manifestations. Early recognition, comprehensive immunological evaluation, and timely renal biopsy are essential for establishing the diagnosis and initiating appropriate treatment, thereby improving clinical outcomes.

## Data Availability

The raw data supporting the conclusions of this article will be made available by the authors, without undue reservation.
